# Electrospun Drug-Loaded and Gene-Loaded Nanofibres: The Holy Grail of Glioblastoma Therapy?

**DOI:** 10.3390/pharmaceutics15061649

**Published:** 2023-06-03

**Authors:** Lynn Louis, Bor shin Chee, Marion McAfee, Michael Nugent

**Affiliations:** 1 Materials Research Institute, Faculty of Engineering, Technological University of the Shannon, Midlands Midwest, Athlone Main Campus, N37HD68 Athlone, Ireland; a00278594@student.ait.ie (L.L.); b.schee@research.ait.ie (B.s.C.); 2 Centre for Mathematical Modelling and Intelligent Systems for Health and Environment (MISHE), Atlantic Technological University, F91YW50 Sligo, Ireland; marion.mcafee@atu.ie

**Keywords:** electrospun nanofibres, glioblastoma (GBM), electrospinning

## Abstract

To date, GBM remains highly resistant to therapies that have shown promising effects in other cancers. Therefore, the goal is to take down the shield that these tumours are using to protect themselves and proliferate unchecked, regardless of the advent of diverse therapies. To overcome the limitations of conventional therapy, the use of electrospun nanofibres encapsulated with either a drug or gene has been extensively researched. The aim of this intelligent biomaterial is to achieve a timely release of encapsulated therapy to exert the maximal therapeutic effect simultaneously eliminating dose-limiting toxicities and activating the innate immune response to prevent tumour recurrence. This review article is focused on the developing field of electrospinning and aims to describe the different types of electrospinning techniques in biomedical applications. Each technique describes how not all drugs or genes can be electrospun with any method; their physico-chemical properties, site of action, polymer characteristics and the desired drug or gene release rate determine the strategy used. Finally, we discuss the challenges and future perspectives associated with GBM therapy.

## 1. Introduction

**Figure 1:**
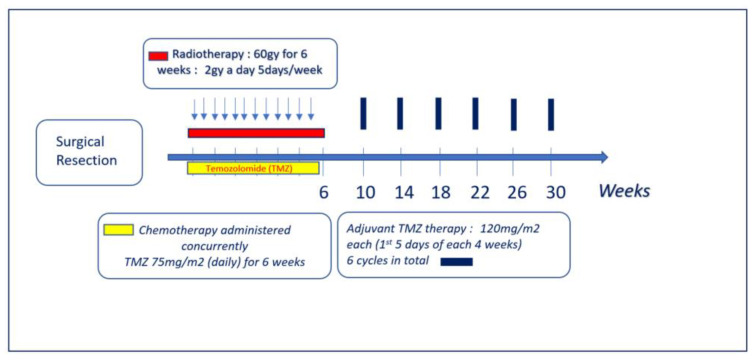
Stupps regimen initial treatment course for newly diagnosed patients with Glioblastoma (GBM)/CC BY 3.0.

Glioblastoma multiforme (GBM) is the most prevalent aggressive primary malignant brain tumour in adults with high infiltrative abilities. The recurrence rate of these tumours remains obstinately high and serves as the primary aetiology of deaths. Tumour recurrence happens within the 2 cm region of the resected margin in 90% of clinical cases. To lessen the nadir when a brain tumour is diagnosed, a combination of radiotherapy and chemotherapy using temozolomide, also known as Stupp’s protocol (Figure 1), remains the mainstay post-surgical resection. The cardinal limitation of chemotherapeutic agents in the treatment of brain pathologies lies in achieving the desired therapeutic outcome due to the blood–brain barrier (BBB) that protects the central nervous system. The BBB impedes the delivery of optimal doses of therapeutic agents to the GBM tumour site—a relationship that can extend to high systemic toxicities associated with the therapeutic agent [1]. While the BBB represents a significant obstacle in drug delivery, pathways that obstruct its permeability are yet to be fully understood. This epistemic gap prevents attempts at effective fabrication of the BBB for drug delivery and currently, only highly invasive treatment where the BBB is disrupted is available. Disruption of the BBB cannot be considered as a long-term treatment [2]. Therefore, the need for novel approaches cannot be overemphasized to understand the biology of GBM and its therapeutic vulnerabilities [3].

**Figure 2:**
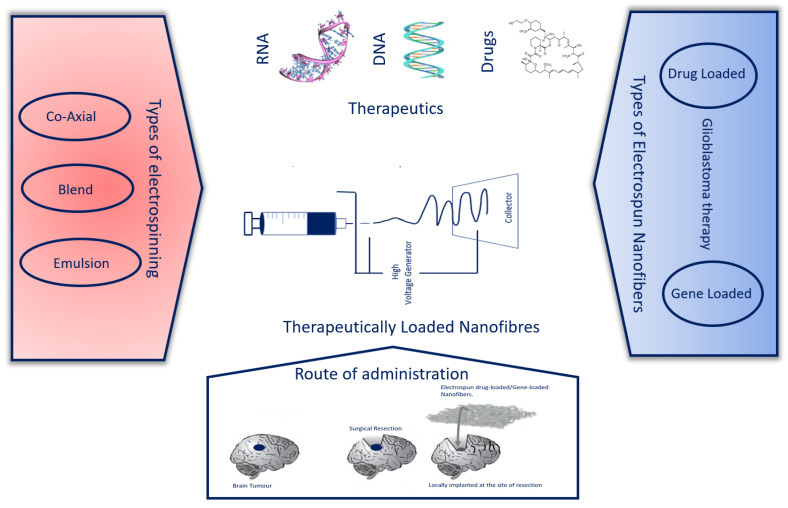
Schematic illustration of the type’s electrospinning and therapeutically loaded nanofibres.

Electrospun nanofibres (Figure 2) for drug delivery have been developed to circumvent these limitations, and have proven to address a major hurdle in GBM therapy, which is the delivery of gold-standard chemotherapeutics via a sustained release while simultaneously preserving the encapsulated drug. This allows the release of the drug at a certain level within a valid therapeutic window to the tumour site, avoiding excessive drug circulation in the systemic circulation. This relationship affords patients long-lasting and curative benefits [4,5]. Electrospinning (ES) is a one-step process that produces small-diameter fibres or ultra-fine fibres in the range of micro- to nanometers. Electrospun nanofibres are valuable materials owing to the high tunability of physico-chemical properties, as well as an adaptable formulation which has been applied in a diverse array of applications in the biomaterial field, particularly in biomedical devices, tissue engineering, and drug delivery [6]. These nanofibres are known to possess a high surface area to volume ratio, which enhances drug encapsulation efficiency, and depending on the chemical composition of the polymer, the electrospun membrane can have properties that respond to a certain stimulus, which can enable control of a drug’s release rate and mechanism [7,8]. A diverse array of anticancer drugs such as Doxorubicin (DXR) [9], Paclitaxel (PTX) [10], Cisplatin (CP) [11], and Salinomycin (SALI) [12], as well as various nanomaterials such as multi-walled carbon nanotubes (MWCNTs) [13], gold nanorods [14], iron oxide nanoparticles (IONPs) [15] and gene ‘drug’ therapy, have been encapsulated in nanofibres to treat GMB and different cancers. Extensive research has been dedicated to the development of ES nanofibres as a vehicle for the integration of various bioactive agents and gene therapy via blending, emulsion and co-axial electrospinning. These advanced electrospinning techniques may facilitate the modulation in the loading and the release of poorly soluble and insoluble drugs.

An alternative to targeted drug delivery would be the powerful technology of gene delivery in GBM therapy. As a preventive or therapeutic measure, gene therapy is employed to regulate gene expression in the altered cells at the DNA or mRNA level by correcting gene transcription and translation processes on the path to recovery [16]. One of the fundamental aspects of gene therapy delivery is the selection of an appropriate gene delivery vehicle owing to the fact that gene-based therapy requires a gene that can be tuned to modify or repair the affected gene at a molecular level [17]. This warrants the developed vehicle to target the selected cells without compromising the healthy immune system or triggering a toxic response. Furthermore, the gene should remain unchanged by preserving the encapsulated gene within the nanofibres while diffusing through complex intracellular barriers. The incorporated gene must be efficiently encapsulated to remain viable and protected from the surrounding enzymes to exert the desired therapeutic efficacy [18]. The goal of altering genetic information is to induce or activate signals to trigger apoptosis in cancer cells [19]. In this instance, the function of triggering apoptosis in gene delivery technology is a requisite in GBM therapy where the results offered are potentially promising to improve the targeted function. The occurrence of glioma is related to the sequential acquisition of genetic alterations; therefore, a gene delivery system serves as an alternative approach to negating the limitations associated with conventional therapy [20]. To increase the potential of gene therapy, the integration of gene delivery systems with electrospun nanofibres is an excellent strategy that can be readily utilized in GBM therapy and various other biomedical applications.

The use of electrospun nanofibres as drug and gene delivery scaffold systems have been reported by various different researchers [18,21,22]. The advantageous characteristics of electrospun nanofibres used as a spatial template for gene delivery include the ability to mimic the extracellular matrix (ECM), the fact that it is a one-step process, the various choices of polymer material, the large surface area to volume ratio and the ability to produce structures of varied physical and chemical properties. Extensive research and time have been dedicated to the successful delivery of gene or nuclei acid carriers to the tumour tissue. In the molecular to nanoscale dimension, a plenitude of carrier-based approaches have been attempted to date, made up of bio-inspired assemblies which are categorized into viral and non-viral vectors. The use of viral vectors has failed to achieve FDA approval due to inefficient tumour penetration and limited efficacy based on clinical trials for GBM [20]. However, non-viral vectors (polymeric and non-polymeric delivery systems) have shown positive results as gene vectors for GBM treatment in pre-clinical studies, with a few non-polymeric vectors entering clinical trials [23]. Motivated by the limitations of the viral counterparts, we discuss the use of non-viral vectors in detail.

This review primarily discusses the different types of electrospinning techniques as a revolutionary technology in the generation of drug-loaded and gene-loaded electrospun nanofibres. Furthermore, we discuss the different strategies employed in the combination of electrospun nanofibres loaded with drug therapy and gene therapy agents to achieve a sustained release, its potential application in GBM therapy and the in vitro and in vivo induced performance of these loaded nanofibres. Finally, we discuss the progress in the current application of these nanofibres in cancer therapy, including the transition from significant research results to approved potential carriers in clinical applications.

## 2. Electrospinning

**Figure 3:**
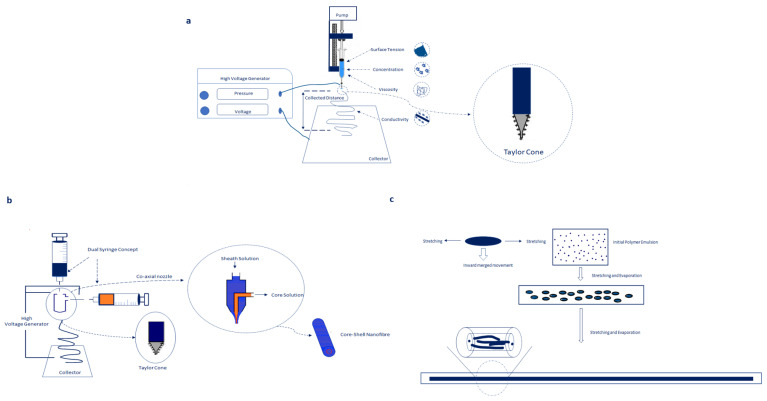
(**a**) Illustration of electrospinning procedure/CC BY-NC 4.0, (**b**) Schematic Diagram of Co-Axial Electrospinning process, (**c**) Schematic Illustration of Emulsion Electrospinning, reproduced with permission from [27].

Electrospinning is a unique and versatile technique that depends on the electrostatic repulsion between surface charges to constantly draw nanofibres from viscoelastic fluids. It produces fibres with diameters that range from less than 3 nm to 6 μm which are able to resemble the ECM and possess the required features to be applied in the area of medicine [24,25,26]. Figure 3a shows a typical electrospinning apparatus which is composed of a high-voltage supply, a reservoir for a polymer solution and a collector plate.

The technique involves a high voltage being applied through a needle that is attached to a syringe. The syringe is filled with the drug–polymer and or/gene–polymer solution, and a strong electricfield between the needle and the collector plate is induced. When the electric field overcomes the surface tension of the polymer solution, a charged jet is emitted from the needle tip onto the collector plate. During the electrospinning process, the morphology and architecture of an electrospun structure can be fabricated and controlled by manipulation of various parameters (applied voltage, pressure, the distance between the needle and collector plate), the polymer–drug solution’s properties, (viscosity, type of polymer, surface tension, surface charge and electrical conductivity) as well as environmental parameters (humidity and temperature) making this technique highly sought after in the area of medicine [8,18,22,28]. The instabilities of the final polymeric solution determine the final architecture of the produced fibres which is a byproduct of the Coulomb force in the charged polymer fluid. The processing parameters are responsible for the final fibre morphology; therefore, it can be tailored and adjusted according to the desired therapeutic outcome. Various polymeric materials such as polymers, inorganic compounds, and hybrid (organic/inorganic) compounds have been electrospun to fabricate ultrafine fibres with diameters in the range of nanometers to micrometres [29,30]. Encapsulation of different drugs such as antibiotics, chemotherapeutic agents and gene therapy such as DNA, mRNA, and proteins has been achieved using electrospinning techniques such as coaxial, blending and emulsion techniques [31,32]. Electrospun nanofibres have been widely applied in drug delivery, tissue engineering, and biomedical applications [33,34].

### 2.1. Co-Axial Electrospinning

Co-axial electrospinning is a technique where two polymeric solutions are simultaneously electrospun [34]. Co-axial electrospun fibres contain two phases: the core and the sheath phase (Figure 3b). The sheath phase protects the drug or gene from harsh environments allowing it to be transported to the tumour site of interest preserved and unaffected [35]. This technique allows for a prolongation of the release time of bioactive agents as well as preventing an initial burst release due to the presence of the shell layer [34]. For smaller molecules, a slower release from hydrophilic materials is enabled. Co-axial electrospinning results in fibres yielding zero-order kinetics for a controlled release [36]. The drug/gene type, degradation rate, and diffusion coefficient of the core/sheath membrane strictly determine its release [37]. When employed for gene delivery, the gene is encapsulated in the core phase of the nanofibres, ensuring that the therapeutic gene is well preserved, preventing a burst release and allowing the gene to be released in a prolonged and sustained manner.

To corroborate this, Sukumar et al. [38] fabricated a composite core–shell nanofibrous scaffold encapsulated with a suicide gene and a pro-drug targeted at cancerous cells. The core–shell nanofibrous scaffold composed of PEO/bPEI was electrospun to encapsulate 5-fluorocytosine (5-FC) within the core. The aim was to achieve a controlled and sustained delivery. Cytosine deaminase::uracil phosphoribosyl transferase CD::UPRT polyplexes were loaded in the shell via co-axial electrospinning. The results obtained showed that the suicide gene exhibited prolonged expression by the cancerous cells and cellular apoptosis being triggered due to the controlled and sustained release observed for the pro-drugs. The surrounding cells were also impacted, confirming the anticancer efficacy of the developed dual delivery system.

In drug delivery, a sustained release/controlled release of the drug can be achieved when the structure and composition of the core phase and sheath phase of the fibre design are controlled. Desirable mechanical properties can be achieved when suitable components are chosen to achieve the desired therapeutic outcome. Co-axial fibres can resemble the tumour microenvironment and be used as a scaffold [39,40]. A biocompatible polymer is essential for the shell, whereas for the core, a less bio-compatible polymer can be selected [2]. An example would be the fabrication of PVA and bevacizumab as well as PCL and gelatin to prepare degradable drug-loaded core–shell nanofibres via co-axial electrospinning by Desouza et al. for age-related macular degeneration (AMD) treatment [41]. In another study, Sakib et al. [42] successfully fabricated PCL loaded with 5-fluorouracil (FU) and Paclitaxel (PTX), recording an improved therapeutic effect in breast cancer treatment compared to the use of the anti-cancer drug alone.

### 2.2. Blend Electrospinning

Blend electrospinning is dependent on the mix of the polymer–drug solution prior to being electrospun to achieve a one-phase electrospinning method [43]. It involves the encapsulation of genetic material or a drug via simple mixing of the polymer. Blend electrospinning can be used to encapsulate hydrophilic and lipophilic drugs as well as biomolecules into the fibre [32]. The release kinetics of blend-electrospun fibres is dependent on desorption or diffusion or the degradation rate of the polymer matrix. Depending on the embedded concentration from the inside of the polymer matrix to the outside, the diffusion rate is determined. For example, a drug diffuses through the polymeric layer when associated with a non-biodegradable polymer. However, the degradation of a system is an extra consideration in the case of a biodegradable polymer. This is because of the swelling of the polymeric matrix from the solvent causing a rearrangement of polymeric chains due to an increase in volume. The diffusion-controlled release applies the same concept; however, the compatibility of the solvent with the drug/gene influences the release via diffusion from the polymer/solvent layer [44].

Therefore, it is crucial to understand the drug release behaviour and the type of polymer, either biodegradable/non-biodegradable, to ensure uniform distribution of the drug within the electrospun fibres [43]. For successful encapsulation of a drug or gene via blend electrospinning, the physico-chemical properties of polymers and their interaction with the drug/gene must be carefully considered, as the drug/gene encapsulation efficiency, drug/gene distribution within the fibres, and drug/gene release kinetics are influenced by them [43]. To simplify, the hydrophilic–hydrophobic drug–polymer characteristics must be compatible. In one study, Zheng et al. [45], studied the use of surfactants and their influence on electrospun nanofibres. The study fabricated poly-(l-lactic acid) (PLLA) nanofibres that were encapsulated with the lipophilic drug paclitaxel (PTX), the hydrophilic drug doxorubicin hydrochloride (DOX), and lipophilic doxorubicin base (DXR) to study the high therapeutic take-up inside the electrospun fibres. Based on the study, the burst release of the drug had a direct relationship with the compatibility of the drug and the polymer in question. The results obtained showed zero-order kinetics as the model of best fit due to the PLLA fibres degrading in the presence of proteinase K [45]. A sustained release can be achieved by the correct blending of hydrophilic–hydrophobic polymers such as gelatin, polyethylene glycol (PEG) and poly(vinyl) alcohol (PVA) or by employing the amphiphilic polymer PEG-b-PLA to increase drug-loading efficiency and prevent a burst release [43].

### 2.3. Emulsion Electrospinning

Emulsion electrospinning is the combination of both co-axial and blend electrospinning with an emulsification approach. The core–sheath structure is formed using a single nozzle. In comparison to blend and co-axial electrospinning, emulsion electrospinning has partially different processing conditions (Figure 3c) [44,46]. Emulsion electrospinning is based on two or multiple phases where no mixing occurs during the electrospinning process [44]. The fibre core is formed from the droplet phase, whereas the sheath is formed from the continuous phase [44,47]. The release kinetics are affected by the core morphology of emulsion nanofibres and the diffusion and degradation mechanisms govern the drug release. In emulsion electrospinning, the formation of the integral core–sheath layer is ideal to alleviate an initial burst release, prolong drug release to ensure the optimum therapeutic effect is reached, and maintain the structural and biological integrity of the drug molecule.

Luo et al. [48], fabricated hydroxycamptothecin (HCPT) into the core of the electrospun fibre and 2-hydroxypropyl-yclodextrin (HPCD) into the sheath by emulsion electrospinning to evaluate in vitro anti-tumour activities on cancer cell lines and intra-tumoural implantation in vivo on tumour bearing mice. HCPT-loaded electrospun fibres in vitro cytotoxicity tests demonstrated 20 times higher inhibitory activity in HepG2 cells than free HCPT during the 72 h incubation period. Hepatoma H22 cells were subcutaneously injected into Kunming mice to form solid tumours for in vivo tests on the antitumour efficacy. Based on the tumour volume, survival rate and body weight changes, HCPT-loaded fibres indicated superior in vivo antitumour activities and fewer side effects compared to free HCPT. HCPT-loaded fibres induced necrosis and apoptosis based on the examination of caspase-3 expression [48].

The recent advances in electrospun fibres have decreased the systemic toxicities associated with bioactive agents used in chemotherapy and provide safe concentrations of the drug at the local tumour site, enhancing bioavailability and preventing tumour recurrence. The remarkable properties of electrospun fibre scaffolds such as high surface area, high surface porosity and interconnected pore networks resemble those of the tumour microenvironment (TME) [18]. These fibre scaffolds are designed to mimic the TME by studying the propensity of the invasion of GBM cells to discern the invasive nature of these cells [49]. The purpose of fibre scaffolds is to better understand the heterogeneity of GBM and ways to formulate anti-invasive therapies [7,50]. Furthermore, aligned nanofibres are developed for tumour cells to invade and to guide cells away from the primary tumour site to an extracortical region [51].

## 3. Release Kinetics of Electrospun Nanofibres Is Dependent on Physico-Chemical Properties of Electrospun Nanofibres

The choice of polymers electrospun into nanofibres has a great influence on controlling gene and drug release. The synergism between the polymer and the release profile is due to the ability to exploit the physico-chemical properties of the polymer to achieve the desired drug release. Aside from the commonly assessed physico-chemical properties, such as fibre morphology, composition and thermal properties, further properties that are often overlooked but are significant in the design of an electrospun scaffold are the distribution and mechanical properties [52]. Herein, we provide a conceptual framework for the required physico-chemical properties of electrospun nanofibres for GBM that influence the drug release kinetics. They include hydrophobicity, hydrophilicity, drug and gene distribution and mechanical properties. The drug/polymer or gene/polymer compatibility is also attributed to the adequate distribution of a drug within the polymeric nanofibres to achieve a sustained release [53]. Both hydrophilic and hydrophobic polymers have their own completely different drug-release mechanisms [54]. Based on the general principle of solubility “like dissolves like”, hydrophobic and hydrophilic drugs can be encapsulated in hydrophobic and hydrophilic polymers, respectively.

For example, in one study, Zheng et al. [45] studied the use of surfactants and their influence on electrospun nanofibres. Fabricated poly-(l-lactic acid) (PLLA) nanofibres were encapsulated with the lipophilic drug paclitaxel (PTX), the hydrophilic drug doxorubicin hydrochloride (DOX), and a lipophilic doxorubicin base (DXR). The main aim of this study was to examine why burst release occurs in electrospun fibres. The DXR base and PTX base showed good encapsulation properties due to their compatibility with the PLLA polymer and solvent used. The release profile of PTX and DXR in the presence of proteinase K at concentrations of 0.01 mg/mL and 3.0 × 10^−3^ mg/mL, respectively, obeyed the zero-order release kinetics which confirms a controlled release. The release of both drugs was mainly due to the degradation of PLLA nanofibres. However, doxorubicin hydrochloride was observed near the surfaces of PLLA nanofibres, causing an obvious burst release. Based on the results observed, it was concluded that the deciding factor for electrospun fibres is the solubility and compatibility of the drug in the drug/polymer/solvent vehicle. The study proved that surfactants have an influence on the diameter of electrospun fibres which, in turn, influences the drug loading capacity.

Another type of polymer studied is peptide nano assemblies, which have shown remarkable properties in sustaining drug and gene release for delivery into tumour cells. Due to their ability to form a wide range of nanostructures, self-assembling peptide hydrogels are used [55]. Lebedenko et al. [56] developed new peptide conjugates by amalgamating the anti-inflammatory antitumour compound azelaic acid with angiopep-2, which efficiently self-assembled into nanofibres. Functionalizing of the two nanofibres was then performed using two known peptides with receptor-mediated recognition, known as A-COOP-K sequence forming supramolecular hierarchical structures, that efficiently encapsulated the chemotherapeutic agent doxorubicin (DOX). The self-assembled polymer showed a drug release profile that is dependent on the concentration of the drug and as well a twofold increase under acidic conditions in the span of two weeks. To mimic the growth conditions of tumour cells, two cell lines were employed—U-87-MG and U-138-MG—for GBM cells grown under serum and serum-free conditions. The cell proliferation abilities of U-87 and U-138 MG of GBM cells were inhibited by the drug-loaded assemblies. Lebedenko and team also developed three-dimensional spheroids of different sizes to mimic tumour cells to evaluate drug efficacy and internalization. Results showed uniform distribution of DOX throughout the grown spheroids and high DOX internalization with emphasis on serum-free conditions. The nano assemblies also showed excellent BBB penetration, rendering it a suitable peptide-based nanocarrier. This confirms that a mechanistic approach for drug delivery is not limited to 2D cell cultures, but also 3D tumouroids that mimic the tumour microenvironment.

In another scenario, to achieve a programmed release, a multi-modal approach by Yang et al. [57] was achieved by the fabrication of micelles assembled from a biodegradable amphiphilic monomethoxy poly-(ethylene glycol)-block-poly(e-caprolactone (mPEG-PCL) copolymer encapsulated withhydrophobic curcumin and subsequently blending micelle powder with the hydrophilic doxorubicin (DOX) in polyvinyl alcohol where this final solution was then electrospun. Based on the two different drugs, within the electrospun nanofibre, a time-programmed release was observed, which allows for temporal and spatial regulation. The tumour cell progression assay revealed that the timely release of multiple drugs has a significant effect on tumour regression by improving chemotherapy efficiency and simultaneously reducing side effects.

Hydrophilic drugs have proven to be difficult when it comes to achieving a proper sustained release and successful incorporation into a nanofibre. Therefore, studies have shown that incorporating hydrophilic anticancer drugs into hydrophobic polymeric fibres has a positive outcome owing to the distribution of the drug in the core of the polymer. Two suitable methods, co-axial electrospinning and emulsion electrospinning, can be utilised, where the drug and polymer are mixed to obtain a uniform solution [58,59]. In another study conducted by Xu et al. [60], the authors fabricated an amphiphilic poly-(ethylene glycol)-poly-(l-lactic acid) (PEG-PLLA) di-block copolymer via encapsulation with water-soluble DOX with a diameter in the range of 300 nm^−1^ μm. The purpose of this study was to embed the hydrophilic drug DOX into a hydrophobic polymer. The fabricated fibres successfully encapsulated the entire DOX drug due to the incorporation of the aqueous solution of DOX into the PEG-PLLA/CHCl_3_ solution as highly immersed emulsion drops. The burst release was significantly reduced compared with suspension-electrospun fibres. The release for the emulsion-electrospun nanofibres was dependent on the combined mechanism of diffusion and enzymatic degradation.

Another factor that should be considered in terms of the release of a drug or gene is the distribution of the drug or gene within the functionalized nanofibre. The distribution of a drug or gene has a major influence on the release profile of the carrier, especially when the physico-chemical properties are manipulated to tailor the drug release. Xu et al. [45] created two different types of distribution patterns of the functionalised polymer with the drug ibuprofen (IBU) within the protein Gliadin. The obtained distribution was homogenous in the monolithic fibres fabricated via modified co-axial electrospinning, and the other was heterogeneously distributed in the core/shell fibre via the conventional co-axial process. The SEM and XRD results concur with each other, showing that the drug and protein were distributed differently in the two different morphologies of the nanofibres. IBU showed an amorphous distribution within the monolithic fibres; however, residual IBU was found in the crystal lattices of the core/shell fibre. Compatibility between IBU and gliadin was observed in the FTIR and RM spectra. The tailored physico-chemical properties showed an influence on the in vitro release, showing that a heterogenous distribution could provide a better-sustained release than its counterpart, which showed an initial burst release followed by a sustained release, confirming that drug distribution has an influence on the release profile.

### 3.1. Electrospun Nanofibres for Hydrophobic Drugs in Cancer Therapy

The delivery of a hydrophobic drug via a polymer matrix of an electrospun hydrophilic carrier remains a challenge due to stability issues of the therapeutic agent causing a knock-on effect on the drug release rate and the concentration of the drug affecting therapeutic response [43,61]. Most chemotherapeutic agents used in the treatment of glioblastoma are hydrophobic in nature with poor water solubility; therefore, the use of nanofibres enhances oral absorption due to their high surface area [61]. Poor solubility and instability of a drug molecule make it difficult to achieve sustained release with a suitable concentration within the desired time period for antitumour agents that are extremely hydrophobic [43]. In one study, Laha et al. [26] fabricated gelatin nanofibres by crosslinking via exposure to saturated glutaraldehyde (GTA) and further encapsulating the hydrophobic drug piperine. The aim was to determine the morphology of the nanofibres, drug stability and crosslinking effects. The results obtained showed that a minimum of 6 min was required to achieve crosslinking with improved thermal stability. Piperine was found to be stable in hydrophilic gelatin nanofibre carriers. The in vitro release study showed that piperine was delivered over a prolonged duration of release. In another study, Liu et al. [62] fabricated a fast-dissolving drug delivery membrane to enhance the fast dissolution of poorly water-soluble quercetin and tamoxifen citrate by electrospinning core/shell nanofibres with ultrathin shells. Zeng et al. [63] studied the encapsulation of the hydrophobic drugs rifampicin and paclitaxel by electrospinning directly into poly-(l-lactic acid) (PLLA) fibres, and the polymer–drug–solvent system showed nearly zero-order kinetics of drug release. Xie et al. [64] investigated the use of electrospun PLGA-based micro and nanofibres as an implant to achieve sustained delivery of PTX in the treatment of G6 glioma. Fibres of around several tens of nanometers to 10 mm were successfully obtained and fibres of around 30 nm were obtained post-addition of organic salts. The paclitaxel-loaded PLGA micro- and nanofibres achieved an encapsulation efficiency of more than 90%, which is confirmed by the in vitro studies where a sustained release was achieved for more than 60 days. The IC50 value of paclitaxel-loaded PLGA nanofibres (36 mg/mL, calculated based on the amount of paclitaxel) suggested that the cytotoxicity test is comparable to the commercial paclitaxel formulation.

It is important to ensure the hydrophobic–hydrophilic properties of the drug and polymer are considered to achieve sufficient encapsulation, drug distribution and release kinetics of the drug [43]. Better encapsulation would mean that the drug is properly dissolved in the drug/polymer/solvent system and it is not just dispersed in the solution. Improper encapsulation causes migration of the drug to the surface of the electrospun fibres during electrospinning, resulting in a burst release. Xie et al. [48,65] investigated this, whereby poly-(d,l-lactic acid)-PEG electrospun nanofibres were fabricated via blend electrospinning using 2-hydroxypropyl-β-cyclodextrin (HPCD) as the solubilizer and loaded with hydroxycamptothecin (HCPT). The in vitro cytotoxicity test results suggest that HCPT-loaded electrospun fibres showed seven times higher inhibitory activity against cancer cells compared to the free drug during the first 72 h of incubation. The blend electrospinning method, however, caused a biphasic release; therefore, emulsion electrospinning was employed to investigate HCPT in the presence of HPCD to obtain core–shell-structured fibres. The blend electrospinning resulted in significantly faster HCPT release and a higher degradation rate than electrospun fibres via emulsion electrospinning. The in vitro cytotoxicity test indicated a 20 times higher inhibitory activity against HepG2 cells than free HCPT during 72 h incubation. Blend electrospinning was able to retain 85% of the drug’s active lactone form within the electrospun fibres, whereas emulsion electrospinning was able to retain 93% during incubation for over 1 month.

### 3.2. Electrospun Nanofibres for Hydrophilic Drugs in Cancer Therapy

The improvement in drug loading of hydrophilic drugs through fabricated electrospun nanofibres has gained much attention in the context of controlled release [66]. Small-molecule drugs classified as Type 1 (hydrophilic) are water soluble and used in the treatment of cancer and many other diseases [67]. Small hydrophilic molecules are rapidly cleared from systemic circulations or the local site of application, decreasing their therapeutic efficacy as well as requiring frequent dosing [68]. Therefore, encapsulation of small hydrophilic molecules can improve the pharmacokinetics and biodistribution profile [68]. However, to date, despite the desired positive features of hydrophobic-polymer-based scaffolds, hydrophilic therapeutic agents pose a challenge due to their solubility properties and the propensity of these hydrophilic drugs to escape these scaffolds, resulting in undesired burst release [69]. These molecules have a weak interaction with many conventional drug carriers such as hydrogels due to their low molecular weight, good water solubility and hydrophilic nature [70]. A drug is considered compatible with a polymer depending on its encapsulation efficiency and sustained release. The tendency of a hydrophilic drug to migrate to the surface of the fibre can be limited by the compatibility of the hydrophilic drug and the polymer. A burst release is inevitable for hydrophilic drugs due to their inability to be fully encapsulated in nanofibres, therefore escaping to the surface of these fibres. Chemical modification of a hydrophilic drug can be conducted by transforming them into a pro-drug that is more lipophilic to enable it to be encapsulated into the lipophilic matrix of a fibre [71]. Therefore, the physico-chemical parameters such as solubility, pKa, lipophilicity, permeability and stability—also known as Lipinski’s rule of five (RO5)—are crucial in designing the perfect drug/polymer/solvent combination [72]. The aqueous solubility, partition coefficient, ionization and pka molecular dipole, glass transition and melting temperature are diverse in small molecule drugs.

These are significant factors that affect the drug–solvent–polymer combination and the final solid dispersion. Model hydrophilic compounds are crucial in determining the structure–function relationship between fibre formulation characteristics and drug release profiles, but the interpretation of these model drugs and polymers should be carried out with caution [73,74]. A sustained release of at least 7 days has so far been achievable and primarily limited to small-molecule or large biological macro-molecule hydrophobic drugs. Hydrophobic drugs are more able to achieve a sustained release due to being poorly soluble, their large size and their partitioning into insoluble polymers [73,74]. Fabrication of polymeric fibres loaded with hydrophilic drugs using conventional electrospinning techniques to achieve a controlled release is still proving to be a challenge due to their distribution on the fibre surface leading to a burst release [75]. Due to these limitations, co-axial, blend and emulsion electrospinning can be employed to gain an in-depth understanding of the sophisticated interplay between different parameters of the drug–polymer–solvent combination. This can then lead to a rational design to achieve the desirable sustained release. The use of co-axial electrospinning, which includes a core and a sheath flow, can employ two different solvent systems simultaneously [76]. In this case, the core phase is used to incorporate the hydrophilic drug which can then be embedded into the hydrophobic polymer in the sheath phase. The sheath phase in this case serves as a physical barrier allowing sustained release of a therapeutic agent. When loading a core and a sheath phase, two different release patterns from one carrier can be achieved.

A dual drug release can also be achieved when the shell of the fibres is embedded with different active therapeutic agents. Simple blend electrospinning involves the process of preparing a drug-loaded fibre whereby the drug is dissolved in one or more polymer-spinning solutions. Subsequently, a single nozzle is used for electrospinning the drug–polymer combination. This method can be used in the encapsulation of hydrophilic and lipophilic drugs which can be embedded into electrospun fibres. Most of the research on the fabrication of electrospun fibres loaded with a hydrophilic drug has been centred around antibiotic and antiviral compounds [74]. The ability to achieve a sustained release with these antibiotics and antiviral therapeutic agents proves that it can be a beneficial approach in the treatment of cancer. Castillo-Ortega et al. [77] fabricated cellulose-acetate poly-(vinyl pyrrolidone) (CA-PVP) loaded with a hydrophilic drug, Amoxicillin, by co-axial electrospinning. The inner spinning polymer was CA, and the outer was CA-PVP. It was observed that the release of Amoxicillin from the fibre’s nuclear layer was pH-dependent, whereby an increase in pH increased the release of Amoxicillin. This is attributed to Amoxicillin being able to form hydrogen bonds with the different components of the fibres at low pH, decreasing release. The release observed was 61% at pH 3 and 79% at pH 7.2, both at 48 h.

Zupancic et al. [78] fabricated poly-(caprolactone) (PCL) loaded with two antibacterial agents, metronidazole and ciprofloxacin hydrochloride, with the aim of developing a prolonged-release drug delivery system for the treatment of periodontal disease. It was observed that the thickness of the PCL fibre’s diameter and its hydrophobic nature prolong the release of hydrophilic drugs, but the rate-limiting step for the drug release was wetting. In this study, it was observed that combination therapy is a beneficial approach in the inhibition of all pathogenic bacterial strains tested in periodontal disease. In another scenario, a twisting method for the generation of nanofibre-based sutures was developed by Chen et al. for the simultaneous delivery of silver and gentamicin via co-axial electrospinning. Gentamicin and pluronic 127 were loaded in the core of the fibres and silver/PCL was loaded in the sheath of the fibres. An initial burst release followed by a sustained release over 5 weeks was observed for silver and gentamicin. The core–shell-loaded sutures were able to kill bacteria more efficiently compared to single-loaded silver and gentamicin fibre sutures, with no impact on the proliferation and migration of dermal fibroblasts and keratinocytes, proving great potential in preventing surgical site infections [79]. Fabricated core/shell nanofibres of poly-(methyl methacrylate)–nylon6 incorporated with Ampicillin through co-axial electrospinning were studied by Shorabi et al. [80]. The drug release mechanism followed a three-stage sustained release throughout a period of 31 days. The initial stage showed a non-Fickian diffusion, whereas stages II and III showed a Fickian diffusion mechanism. Table 1 lists the mechanisms of hydrophobic and hydrophilic drug release based on the type of polymeric drug delivery system.

### 3.3. Mechanical Properties of Engineered Electrospun Scaffolds in GBM

To ensure the integrity of the regional tissue microenvironment, the mechanical properties of engineered scaffolds must match the normal tissue morphogenesis [96]. The stiffness of the brain tissue is usually used as a yardstick when discussing mechanical mismatch between engineered biomaterials and brain tissue. However, bar mechanical properties, there are other essential properties such as tensile strength, viscoelasticity, adhesion and cytoskeletal properties that contribute to solute diffusion which should be characterized more thoroughly [97]. Therefore, in GBM, for effective amalgamation with the host tissue, the mechanical properties of biomaterials implanted into the central nervous system are crucial. However, to date, the ability to reproduce the biomechanical properties of the host tissue and assess the stiffness of the central nervous system tissue and soft biomaterials remains a challenge. In the brain, cellular and tissue stiffness properties define the tissue mechanics as well as the stress transmitted by fluids, which include cerebrospinal fluid (CSF) dynamics and interstitial fluid pressures (IFP) [98]. In the GBM microenvironment, the tissue mechanics denote the solid stress due to an infiltrative growth pattern, where the surrounding ECM and tissue architecture are the main contributors as well as the glioma cells situated in the tumour microenvironment in the cellular compartment [98].

In light of understanding the cardinal manifestations of cancer, six biological capabilities were delineated as the hallmarks of cancer by Hannahan and Weinberg [99]; they include the ability of the tumour to remain undetected by the immune system allowing it to proliferate in an uncontrollable and infinite manner (which translates to the cells possessing sufficient growth signals rendering them insensitive to growth-inhibitory signals bypassing apoptosis), the invasion activation and spreading of tumour cells from the primary site to a secondary site within the host’s body, also known as metastasis, reprogramming of the energy metabolism prior to becoming cancerous, allowing the survival of these cells in harsh conditions of the TME, where cells focus on survival when morphing into cancerous cells, promotion of inflammation—a relationship that extends to the tumour growth capabilities—and the evasion of the immune system which is responsible for the destruction of tumour cells. As research on the characteristics of cancer cells is extensively ongoing, more hallmarks have been identified, in particular, the rigidity of the ECM [100]. However, the cardinal trait of GBM is the stiffness of the ECM, and research has shown that the stiffness of the ECM has an extensive influence on cell morphology and behaviour [100,101,102,103,104]. Lately, the mechanical properties of the ECM have come to the forefront of cancer research, as it has shown to be a major influence on cancer cell behaviour bar chemical signals with particular emphasis on rigidity.

Motivated by their counterparts, Nia et al. [103] proposed four additional physical traits of the conceptual framework of cancer, which include solid stress, interstitial fluid pressure, stiffness and altered microarchitecture. Mechanical forces, which arise from solid stress, are divided into three categories: compressive, tensile and shear [100,103]. These forces are incorporated and transmitted by the ECM [103]. The stiffness of the ECM is a major contributor to metastasis, metabolism, tumourigenesis, and immune response [105]. In GBM, the solid stress is reported to range from <100 pa to 10,000 pa in pancreatic ductal adenocarcinomas [103]. Tumour tissue and cellular properties are two key influences of solid stress in GBM. For example, an increase in tissue volume is the by-product of infiltration, proliferation and matrix deposition [103]. Therefore, the natural viscoelastic structure of the tumour is disrupted by the added volume causing solid stress. Solid stress can also be influenced by the initiation of the host’s solid stress by spatial and geometric factors based on the alignment of the cellular cytoskeletal components parallel to the components in the ECM matrix, or the ‘jamming’ mechanism where a critical cell population is reached, increasing cumulative stress due to overcrowded cells and force [103,106,107]. A disruption of fluid homeostasis in normal organs due to tumour abnormality includes hyperpermeable blood vessels and compressed lymphatic and blood vessels secondary to solid stress [103]. As a consequence, leaky tumour vessels result in an interstitial pressure increase [98]. Another cause could be hydrocephalus, also known as brain swelling, which contributes to increased intracranial pressure resulting in increased interstitial fluid pressure at a tissue level. Stiffness, on the other hand, is defined as the resistance to deformation as a result of applied force [103]. In tumours, stiffness is the most common mechanical deformation which promotes the progression of brain tumours [100,108]. It can also be used to describe the tumour microenvironment, individual cells and the ECM components.

The emphasis on mechanical properties could be a novel approach in terms of the design of electrospun scaffolds in the detection, prevention and treatment of this deadly disease. The use of electrospinning in this case can fabricate highly efficient bio-engineered scaffolds with tailored mechanical properties with diameters in the sub-micron to micron range [109]. Mechanical properties of electrospun fibres include viscoelasticity, yield point stress and strain, relaxation time and total and elastic tensile modulus, and loss of energy with an increase in strain [109]. In biomedical applications, it is crucial that the nanofibrous matrix, when used as a scaffold, is fabricated to possess well-tailored mechanical properties. These scaffold properties are then able to defy the harsh mechanical environment of the TME, which includes tumour progression via the epithelial-to-mesenchymal transition and the increased stiffness of the TME which may be caused by higher density of the tumour cells, increased matrix deposition in cells and increased interstitial fluid pressure. In cancer progression and tissue response, the local tissue architecture plays a cardinal role regardless of solid stress, fluid stress and stiffness. Based on previous research, cancer hallmarks are regulated by the tissue mechanics in glioma [98]. Therefore, to recapitulate this environment, the use of biomaterial scaffolds has shown to be more relevant physiologically compared to the conventional 2D culture system [110].

The effects of electrospinning process parameters on the mechanical properties of polycaprolactone and nanohydroxyapatite were delineated by Doustgani et al. [111]. In the fabrication of nanofibres, the difficulty lies in the handling and low load measurement for the deformation of tensile properties. Therefore, Doustgani et al. experimentally evaluated the process parameters by using the response surface methodology in the design of the experiment for the four different factors of solution concentration, voltage, spinning distance and flow rate. The mechanical properties were then correlated with these variables using the third-order polynomial function. Based on the optimisation study, it was found that with an initial increase in hydroxyapatite, the tensile strength increased; however, with a further increase in concentration, a decrease in tensile strength was observed. At the studied concentration range, the module increased continuously. The mechanical properties of the electrospun fibres decreased with spinning distance where the shorter the spinning distance, the stronger the fibre formation. In terms of voltage application, an improvement in mechanical properties was observed with increased voltage; however, the flow rate had no significant effect on the fibre’s mechanical properties.

Similar to this, an efficient machine-learning approach was attempted by Sarma et al. [112], where a machine-learning model was developed known as the Electrospun Fiber Experimental Attributes Dataset (FEAD) via the collation and development of experimental data from the literature and new features, as well as data from their own experiments. Sarma et al. created this unified conceptual framework to understand the structure–property relationship of electrospun fibres which is central to the development of a device. The polymer employed was polyvinylidene fluoride (PVDF), and the key parameter in controlling the electrical and thermal properties of this polymer was the fibre diameter. This polymer was then modelled against a cornucopia of solutions and electrospinning process parameters using the multi-modal learning approach augmented by a model-agnostic interpretable game-theoretic approach to decipher the relative and absolute relationship between the variables. The experimental data yielded four impactful variables for modelling fibre diameter: feed, polymer concentration, Flory–Huggins chi parameter, and relative energy difference. The developed model was able to generalize the structure–property relationship of any PVDF-polymer-solvent combination, thus promising an effective solution for reducing expensive lab testing to develop the desired mechanical and thermal properties of PVDF fibres.

To ensure mechanical integrity, Kaplan et al. [11] fabricated nanofibre-loaded meshes with cisplatin using electrospinning. The two different biocompatible polymers employed in this study were polycaprolactone (PCL) and poly(glycerol monostearate-*co*-caprolactone) (PGC-C_18_) to ensure the mechanical properties are well suited for dynamic tissues such as the lung. The meshes exhibited super-hydrophobicity based on the rough nanostructure and hydrophobic polymer composition, which translates to a non-wetting nature. These properties were able to sustain the release of cisplatin in a linear fashion over 90 days. The demonstration of the anti-cancer efficacy was conducted using the in vitro Lewis lung (LLC) carcinoma cell assay. The ability to prevent local cancer recurrence by the cisplatin-loaded superhydrophobic meshes was demonstrated in vivo using a murine model that underwent LLC surgical resection. The results showed a statistically significant increase (*p* = 0.0006) in median recurrence-free survival to >23 days in comparison to standard intraperitoneal (i.p) cisplatin therapy of equivalent dose. The i.p cisplatin dose had a marginal effect on preventing local recurrence (8 versus 6 days for no additional treatment, *p* > 0.05). To conclude, the fabricated mesh via electrospinning improved the mechanical flexibility, resulting in strong and yet compliant meshes.

The structure and morphologies of electrospun fibres or scaffolds largely influence their mechanical properties. By varying the processing parameters, we can develop electrospun nanofibres with tailored mechanical properties specific to their applications. The actively ongoing research on electrospinning setups and the ability to develop various fibre assemblies in a more aligned or ordered way opens up new possibilities to tailor fibres with desired mechanical properties.

The influence of the mechanical microenvironment of cancer on cells is profound. The role of cells in this case is to translate information into signals from the mechanics of their substrates. This is a process known as outside–in or mechanotransduction [101]. Electrospun nanofibres have been employed to exploit cell migration due to their ability to mimic the extracellular matrix. The migration of cells is guided and promoted mainly by aligned nanofibres owing to their enviable physical properties such as modulus, size and surface chemistry [113]. The ability of nanofibres to tailor cell migration has enabled them to serve as a promoter of tissue repair and also to help eradicate tumours in vivo [113]. Nanofibres that are employed as substrates can act as a regulator for the migratory behaviour of cells via the nanofibre modulus. For example, Rao et al. [114] developed an aligned nanofibre biomaterial via co-axial electrospinning to study the mechanical and chemical influences on cell adhesion and migration. These models were developed to mimic white matter tract. Different polymers were employed to investigate the influence of chemistry and mechanics on GBM via gelatin, poly(ethersulfone) and poly(dimethylsiloxane in the core, while the shell was composed of common polycaprolactone (PCL) for surface chemistry conservation. The observed results showed that GBM sensitivity was strongly dependent on nanofibre modulus, confirming that the selected materials were suitable based on single-cell morphology, migration speed, focal adhesion kinase (FAK) and myosin light chain 2 (MLC2). In a similar manner, in the shell region, the materials present in the extracellular matrix such as (hyaluronic acid (HA), collagen, and Matrigel) were used to harmonize nanofibre chemistry and the core was composed of PCL to conserve mechanical properties. The results revealed a negative effect on migration, confirming the sensitivity of GBM to HA.

In terms of surface chemistry controlling the migration of cells on fibres, Shin et al. [115] studied the migration of human mesenchymal cells from the peripheral region towards the centre on radially aligned fibrous scaffolds coated with polydopamine with random nanofibres as a control. The surface of the fibre modified with polydopamine improved the adhesion and distribution of the mesenchymal cells without altering the initial cell orientation. This led to fast and directional cell migration finally modulating tissue regeneration.

In another study, Unal et al. [95] studied the cell adhesion properties of GBM. This was conducted via the fabrication of a polycaprolactone (PCL)/gelatin (gel) functionalized with bacterial cellulose nanocrystals (BCNC) via the electrospinning technique in an attempt to mimic the extracellular matrix of GBM. To increase fibre diameters, the concentration of BCNC suspension was increased and the observed morphology of the fibre matrix changed from smooth to beaded with an increase in BCNC suspension. U251 MG GBM cells were employed to determine the in vitro viability of the scaffold, and the cell adhesion and proliferation were also compared with the control PCL/Gel. An enhancement of axon growth and elongation was observed with the PCL/Gel/BCNC which ensures communication between cells and the TME, which triggers tumour recurrence. These results confirm that PCL/Gel/BCNC is suitable for use as a biomimetic GBM tumour platform.

In terms of soft biopolymers, Jiang et al. [116] crosslinked a gelatin/alginate matrix to encapsulate MDA-MB231 triple-negative breast cancer cells and IMR-90 fibroblast cells at a specific initial location relative to each other. After 7 days, multicellular tumour spheroids (MCTS) begin to form due to the co-culture of the MDA-MB-231 cells with an observed increase in size and frequency. Migration of the IMR-90 stromal fibroblasts cells after 15 days was observed through a non-cellularized region of the hydrogel matrix infiltrating the MDA-MB-231 spheroids forming a mixed MDA-MB-231/IMR-90 MCTS, providing a proof of concept that the bio-printed models result in an MCTS that can be maintained for several weeks.

### 3.4. Release Kinetics of Drug-Loaded and Gene-Loaded Nanofibres for Cancer Therapy

Electrospun nanofibres have a proven ability to perform in the field of cancer therapy owing to their multifarious design, ability to release a combination of encapsulated drug/gene, low toxicity, reduced immune response and encapsulation of a cornucopia of poorly soluble drugs and gene–drug therapy [22]. These fibres possess unique physico-chemical characteristics such as fibre morphology, fibre composition and thermal properties [52]. The use of electrospun nanofibres as a drug delivery vehicle was pioneered by Kenawy et al. [117], where the drug Tetracycline was embedded in electrospun fibre mats. The release of tetracycline from poly-(lactic acid) (PLA), poly-(ethylene-co-vinyl acetate) (PEVA), or from a 50:50 blend of the two polymers showed a sustained release over 5 days. To date, there are various chemotherapeutics that have been embedded into electrospun fibres, such as small molecule drugs, antibiotics, proteins, DNA, siRNA and oligo/polypeptides [73].

When designing a drug delivery system in the treatment of brain tumours, the primary step is to understand the TME, which contains malignant cells, and how its dominant role is to regulate tumour formation, progression, and metastasis to defy these harsh environments [118,119]. A decrease in pH in the TME results from glycolysis combined with reduced removal of acidic metabolites [120]. Glucose-dependent adenosine triphosphate (ATP) is produced by the glycolysis process, a hallmark of cancer, and for macro-molecule biosynthesis, glycolytic intermediates are produced [120]. The effect of substrate topography on GBM migration is confirmed by the number of cells dispersed in the white matter compared to the grey matter, despite the resemblance in the ECM molecules in both areas [121]. Therefore, when developing effective therapeutic targets, the interaction between tumour cells and the extracellular matrix and targeting-cell-intrinsic pathways can be used as a benchmark. Local residual tumour and circulating tumour cells post-surgical resection may still give rise to tumour recurrence. Adverse surgical stress on the tumour defence mechanism, unintentional seeding of tumour cells during resection and the impact of anaesthesia are some of the known causal factors of tumour recurrence [122]. In line with these issues, combination therapy, which includes radiotherapy and chemotherapy, is required. However, the uncertainty associated with chemotherapy, such as systemic toxicities, poor bioavailability due to the BBB impeding treatment, and its highly invasive nature, requires the development of localised drug delivery systems. Localised implantable nanofibres have demonstrated reduced systemic toxicities associated with chemotherapy, an ability to circumvent the BBB and prevention of tumour recurrence [123]. Many polymeric systems in research have been embedded with Paclitaxel [124], Temozolomide [88], Doxorubicin [125], and Salinomycin [12] and have been reported in experimental models; however, the critical challenge of prolonged delivery for more than 4 weeks within the intracranial tumour microenvironment remains. Therefore, it is crucial to select an appropriate polymer to enable researchers to tailor the physico-chemical properties such as hydrophobicity, hydrophilicity and mechanical stability which influence the fibre morphology and release kinetics of the encapsulated therapeutic agent.

In one study by Nourouzi et al. [12], Salinomycin (SALI)-loaded nanofibres by direct blending for glioblastoma therapy were developed. Salinomycin has recently been introduced as a novel alternative compared to traditional chemotherapeutic agents. It is an antibacterial and ionophore anticoccidial therapeutic drug. In this study, SALI-loaded poly-(lactide-co-glycolide) (PLGA) was electrospun to fabricate sub-micro (PLGA NFs 165 ± 42 nm and PLGA NFs + SALI 170 ± 57 nm) fibres. The fibres showed well-interconnected porosity, which is beneficial in hampering cell migration and metastasis by capturing cancer cells. The PLGA-loaded SALI formulation at pH 6 and 7.4 showed a similar time-dependent release profile of SALI over 14 days. Over the first 4 days, a rapid release was observed, where ca.80% of the drug was released due to diffusion of the drug at the surface of the nanofibres. Subsequently, a slower release of the remaining encapsulated SALI was observed over the next 10 days due to the degradation of PLGA. The release kinetics and degradation profile followed the Siepmann–Peppas model. This indicates that the mechanism controlling SALI release in the first 4 days was due to anomalous transport, and the initiation of bulk polymer degradation was attributed to the release of encapsulated SALI over the remaining period. Cytotoxic studies were performed on U251 GBM cells and the results revealed that NFs + SALI-treated cells were effective in killing U251 GBM cells with improved cytotoxicity compared to SALI on its own. This is due to the gradual release of the encapsulated drug from the fibres over a period of 48 h. The superiority of SALI release from fibres over systemic SALI administration in terms of apoptosis was also reported. Gene expression studies of NFs + SALI showed an increase in tumour suppressor genes (RBI 1 and RBI 2) as well as the caspase 3 (apoptosis) gene that induces caspase-dependent apoptosis at the same time, showing a decrease in the (wingless/integrated) WNT signalling pathway.

Ranganath et al. [124] successfully fabricated PLGA poly-(lactide-co-glycolide) fibres loaded with Paclitaxcel (PTX), where the main aim was to deliver chemotherapy post-surgical resection. The PTX-PLGA fibres were fabricated with sub-micro and microfibres at (930 ± 35 nm; 50:50 lactic acid:glycolic acid) and (3.5 ± 0.32 µm; 85:15 lactic acid: glycolic acid), respectively, with different molecular weights. The in vitro release profile from both devices confirmed a sustained release of PTX in vitro over 80 days. A higher release rate was observed in the sub-micro fibres than in the microfibres due to the higher degradation rate of PLGA in the higher concentration of glycolic acid. In animal studies, inhibition of tumour growth was demonstrated in C6 glioma cells in BALB/c nude mice. These mice were inoculated with tumours and treated with implanted fibre discs and sheets, showing a decrease in tumour size on days 24 and 32 in comparison to the placebo and PTX control.

In another study conducted by Ramachandran et al. [88], a localized nano-implant was developed for the controlled release of the chemotherapeutic drug Temzolomide (TMZ) in an orthotopic brain tumour in rats. A TMZ-loaded PLGA-PLA-PCL blend was electrospun to form a 3D composite nanofibre implant with different release kinetics, where the drug release was prolonged for one month. Two types of implants were designed: TMZ-FR (fast release) (20 wt% TMZ-loaded wafer designed for 7-day release) and TMZ-SR (slow release) (20% TMZ-loaded wafer designed for one-month release).

**Figure 4:**
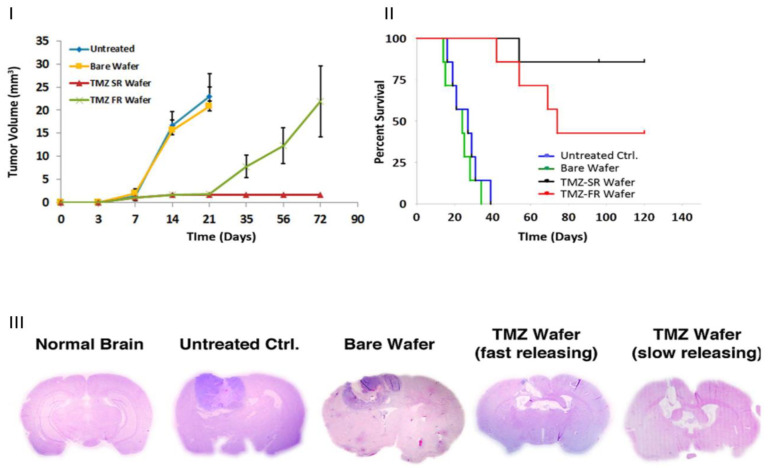
TMZ wafer’s ability to reduce orthotopic C6 Glioma tumours in vivo in rats. Graph (**I**) shows orthotopic C6 Glioma models treated in different groups with a reduction in tumour volume obtained from MRI. (**II**) Kaplan–Meier survival curve showing survival of animals treated in different groups. (**III**) H&E (upper three panels) and Ki67 staining (lower two panels) of brain sections of different treatment groups at day 14/CY BY 4.0 [83].

A 100% release was achieved for TMZ-FR over 7 days, while TMZ-SR achieved a 30-day release, where the first 41% was released in one week, followed by 59% and 80% release in the second and fourth week, respectively. The C6 glioma cell was treated with the 3D-implanted wafer, which showed a constant drug release (116.6 mg/day) with minor leakage into the peripheral blood (<100 ng). This signifies a ~1000-fold differential drug dosage in tumours versus peripheral blood. The implant designed for a one-month release profile increased the survival rate to 85.7% in animals (>4 months) in comparison to the implant with a 7-day release profile, where after 60–72 days, 57.14% of animals treated with TMZ-FR showed tumour recurrence at the tumour site or adjacent to the primary tumour. This indicates that a combination of nanofibres with bulk degradation profiles is able to control the release of chemotherapeutic agents for prolonged periods, hampering tumour recurrence (Figure 4).

Similarly, Ranganath et al. [83], developed a fabricated electrospun PLGA nanofibre embedded with PTX with micro- and nano-structures for intracranial implantation. Malignant glioblastomas in BALB/C mice were treated with this intracranial implant and showed a sustained release of PTX and an enhanced therapeutic penetration 42 days post-implantation of this fabricated device in the mouse brain at about 5 mm from the implant site. Tumour inhibition and low tumour proliferation in the intracranial human GBM cell (U87 MG-luc2) of about ~30-fold was observed after 41 days of treatment. These fabricated sub-micron and nano-scale implants prove that the pharmacokinetics of PTX in GBM tumours is effective in inhibiting tumour proliferation in GBM implanted in mice, and therefore could be a potential regimen in treating highly recurrent GBM.

In radiotherapy, the use of protons, also known as heavy-ion therapy, kills tumour cells by damaging the DNA [126]. This is achieved by directly using charged particles instead of X-ray or gamma radiation, which is also known as photon therapy. Therefore, to inhibit DNA repair pathways, the combination of proton therapy with an electrospun scaffold encapsulated with a chemotherapeutic agent or functionalized with a radiosensitizer will likely be greater than traditional therapy [127]. The advantage of proton therapy is that as it enters the body and makes its way through the tissue, it is able to reserve energy and release it at the site of action, known as peak energy deposition or the Bragg peak. The dose deposition before the Bragg peak is estimated to be 30% of the maximum dose [126]. However, the drawback of traditional photon therapy is that energy is released throughout its pathway to the desired site in the body. The deposition of the photon dose is limited with increasing depth due to energy dumping close to the entrance of the tissue and along the travel path before reaching the desired tissue. Approximately 60% of the dose is lost prior to reaching the targeted site [126]. Therefore, the rationale behind the combination of electrospun scaffolds and radiotherapy is that lesser side effects will be experienced in an appreciable patient population and the synergistic effect of radio-chemotherapy on tumour and tumour metastasis will be increased. When these two treatment plans with different abilities are combined, the therapeutic efficacy is enhanced and the surrounding healthy tissues are exposed to fewer radiation side effects, prolonged release of the encapsulated chemotherapeutic agent at the tumour site, and induced mitotic catastrophe. However, it is important to ensure that the electrospun scaffolds are able to withstand radiation therapy.

For example, Cassan et al. [128] investigated irradiation sterilization of PCL fibre mats to be approved for human implantation. Two different doses, 25 and 33 kGy, were tested using electron-beam (β-irradiation), gamma and X-ray irradiation to determine the mechanical, chemical, thermal and crystalline properties of the fibre mats. Based on the observed results, irradiation caused a decrease in molecular weight, but a significant increase in crystallinity. No major changes were observed in the physico-chemical properties. Cassan et al. hypothesised that the unaffected physiochemical properties are due to a balance between the decrease in molecular weight and the increase in crystallinity. It was also observed that the effects of irradiation are dose-dependent. A higher irradiation dose showed stronger changes. Based on the research conducted, gamma radiation was the least-suited method and β-irradiation exerted the lowest impact.

In terms of the encapsulation of radiopharmaceuticals, Chang et al. [129] fabricated polyethylene glycol (PEG)-liposome-encapsulated ^188^Re nanoparticles to treat human head and neck squamous cell carcinoma (HNSCC). The observed results showed that the three human HNSCC cell lines showed different results. FaDu cells and SAS cells were completely destroyed; however, OECM-1 cells showed no significant effect. An orthotopic tumour model was established in immune-deficient nude mice using FaDu cells (harbouring luciferase reporter genes) implanted in the buccal positions to determine the response of HNSCC to a high dosage of the ^188^Re liposome in vivo via repeated IV administration. Increased accumulation of the ^188^Re liposome in the tumour lesion of nude mice was observed following repeated doses, delaying tumour growth and prolonging survival of mice via Cerenkov luminescence imaging (CLI). These observations were based on the loss of two particular markers, the Ki-67 proliferative marker and the epithelial-mesenchymal transition marker, in resected tumours. No change in body weight was observed following different doses; however, repeated doses saw a decrease in blood counts when compared to a single dose. The pharmacokinetic analysis showed that the circulation of the ^188^Re was prolonged, and an increase in distribution analysis showed the accumulation of these liposomes in tumour lesions and bone marrow following repeated doses. The absorption of a repeated dose was twice the amount compared to a single dose for a 1 g tumour. These data conclude that encapsulated ^188^Re-liposomal radiotherapy is a potential delivery system considering its ability to suppress tumours and prolong survival and systemic circulation with no observed toxicity from repeated dose administration. The ability to encapsulate radiopharmaceuticals into liposomes paves the way for methods such as electrospinning to further magnify its ability as a novel carrier and to overcome the resistance of gliomas to radiotherapy due to intratumoural hypoxia.

Extensive studies have observed that when autophagy of glioma promoter cells is activated, the resistance to radiotherapy can be overcome [130]. For a combination of radio and chemotherapy, currently, nano-radiosensitizers are used in combination with radiotherapy owing to their high concentrations at the tumour site, allowing effective penetration and retention; therefore, the targeting effects are improved. The use of high-atomic-number nanoparticles such as silver, gold and bismuth is the main focus in the design of nano-material-mediated sensitization to achieve an enriched radiation energy deposition in cells [127].

For example, a polydopamine (PDA)-coated Ge11-peptide-conjugated iron-oxide nanoparticle (Ge11-PDA-Pt) encapsulated with cisplatin was fabricated as a carrier by Yang et al. [131] based on ultra-small superparamagnetic iron oxide nanoparticles coated with polyacrylic acid (PAA@ USPIOs). The PAA coating on the USPIOs enriched the cisplatin loading via complexation of the carboxylic acid groups on PAA with the activated chemotherapeutic agent cisplatin. Further surface functionalization was carried out via PDA loading after drug loading, providing photo-thermal properties to the nanoparticles, but not restricting the release kinetics of the drug or iron ions. The observed results showed synergism between radio-chemotherapy under in vitro hypothermia conditions. In another scenario, a therapeutic nano platform designed by Yu Fan et al. [132] was fabricated via the complexation of pyridine (Pyr)-functionalized fifth-generation (G5) polyamidoamine dendrimers with Cu^2+^. These dendrimers were used for radio-enhanced T1-magnetic resonance imaging (MRI) as well as harmonizing radiotherapy and chemotherapy for tumours and tumour metastasis. These positive developments in research on the treatment of glioma are gradually increasing with the development of polymer nanomaterials. Therefore, the combination of electrospun scaffolds with chemotherapy and radiotherapy via functionalization with radiosensitizers is a significant breakthrough in combination therapy.

**Figure 5:**
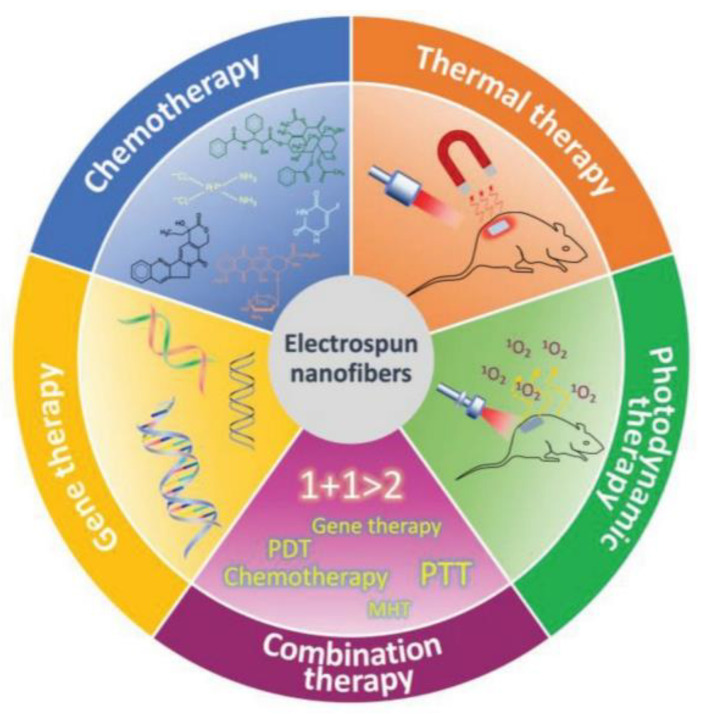
Types of application of electrospun nanofibres used in cancer therapy, reproduced with permission [5].

In cancer, the recurrence of tumours post-surgical resection is a major concern and the low specificity of anticancer agents that destroy healthy tissues and cells is a barrier that is yet to be overcome. Therefore, the use of drug-loaded electrospun nanofibres has proven advantageous in preventing tumour recurrence as well as ensuring a sustained release of therapeutic agents to a cancer-specific hallmark/site. Other drug delivery systems such as micro/nanoparticles run the risk of being metabolized by the spleen or liver, decreasing treatment efficiency. Electrospun nanofibres have been employed for various different applications, for example, gene therapy, photodynamic therapy, thermal therapy and combination therapy, following the success of employing nanofibres in anticancer drug delivery (Figure 5). Nanofibres, however, can be directly implanted at the site of interest, circumventing systemic circulation and the blood–brain barrier.

In terms of gene encapsulation, the ability to tune the physico-chemical properties of an electrospun nanofibre allows for the release kinetics of the loaded gene to be controlled and modulated, inducing a therapeutic outcome according to the intended application. These fibres’ characteristics allow for the preservation of a gene, making them resistant to nuclease degradation. They also mean that the fibres have the ability to control dosing, achieving a sustained release of plasmid DNA, and subsequently gene expression. An example would be blend electrospinning, where the genetic material of interest is embedded within the fibre’s matrix via a simple mixing technique with a polymer cocktail. The solution is then electro-sprayed to form electrospun gene-loaded fibres. The incorporation of a gene vector within the electrospun nanofibre was pioneered by Luu et al. [133], where the gene vector was loaded within the electrospun nanofibre via blend electrospinning using a Tris EDTA buffer containing plasmid DNA, which encoded β-galactosidase driven by the cytomegalovirus (CMV) promoter. Subsequently, the modified EDTA buffer was mixed with the block-copolymers of PLA and poly-(ethylene glycol) (PEG) dissolved in N, N-dimethyl formamide. The plasmid DNA activity was maintained during the fibre formation process. The release of the DNA plasmid was sustained over a 20-day study period, where it was observed that maximum release was achieved within the first 2 h based on the polymer–gene cocktail blend, followed by high levels of gene expression 48 h post-transfection. The DNA release observed was intact and capable of cellular transfection, successfully encoding the β-galactosidase protein.

In another scenario, Nie et al. [134] fabricated PLGA/Hydroxyapatite (HaP) with pDNA (encoding BMP-2)/chitosan (CS) nanoparticles, improving cell attachment, viability and transfection in hMSC cells, with no effect on the biological activity of the DNA or cell activity. Leveraging this, Karthikeyan et al. [135] studied the delivery of siRNA through zein nanofibres to preserve the protein-based fibres to sustain the release of the encapsulated genes within the nanofibres. The in vitro results obtained confirmed that the fabricated zein nanofibres were able to preserve the integrity of the siRNA through a physical interaction achieving a sustained release. The well-preserved gene induced a gene-silencing effect and successfully promoted transfection of siRNA, producing the desired therapeutic outcome.

In another study, He et al. [111] functionalized a biodegradable nanofibrous gene-activated matrix (GAM) via immobilization of a non-viral vector consisting of DNA (pVEGF)-loaded PLGA/PEI nanoparticles modified with cell-penetrating peptide KALA onto a polydopamine-coated electrospun alginate nanofibrous scaffold for skin wound healing. The GAM enabled a sustained gene release and long-term transgene expression of VEGF in vitro based on the series of in vitro analyses conducted to examine DNA release behaviour, degradation properties, transfection efficiency and VEGF expression. Twenty-one days post-implantation in model rats with full-thickness excisional skin wounds, the in vivo results demonstrated significant wound healing properties of the fabricated GAM vehicle, which was able to promote re-epithelization, and in turn, reduce inflammatory responses and increase neo-vascularization.

Co-axial electrospinning was employed by Saraf et al. [112] to produce a core–sheath nanofibre consisting of two solutions where the core consisted of the aqueous solution of PEG embedded with a pDNA and PCL sheath loaded with a PEI-HA (hyaluronic acid) non-viral gene delivery vector. The main parameters in co-axial ES were studied considering how it impacts the morphology of the fibres, its release kinetics and transfection efficiency (e.g., the polymer concentration of both employed polymers (PCL and PEG), the molecular weight of PEG and the effects of plasmid concentration). The demonstrated results confirmed that the investigated parameters directly impacted the fibre diameter; however, the release rate was not significantly impacted. The main factor impacting the release kinetics was the concentration of pDNA. The changes made to the co-axial parameters significantly influenced pDNA and PEI-HA, which resulted in significant EGFP gene expression over 60 days.

Other factors attributed to the regulation of the release profile in electrospun nanofibres are environmental factors that contribute to the degradation of a polymer matrix. The environmental/release profile relationship enables the release of gene vectors from the inner space of a nanofibre. The gene release boils down to a polymer’s degradation pattern, which is divided into surface erosion or bulk degradation. The degradation pattern of a polymer can determine if a burst release or sustained release influences the release kinetics of a gene vector. To accelerate enzymatic degradation, the addition of proteinase K into the release buffer helped to degrade the polyester-based biodegradable polymers, such as poly-(L-lactide-co-ϵ-caprolactone) (PLCL) or poly-(caprolactone) (PCL), resulting in a rapid burst within a short period [18].

Achille et al. [113] fabricated ES scaffolds using polycaprolactone (PCL) alone working as a control and PCL with plasmid DNA encoding for either Cdk2 (Cdk2i) and EGFP (EGFPi acted as a control) shRNA. The ES fibres remained intact for more than two weeks in a physiological buffer. However, during the third week of incubation, degradation was visible. Over 21 days, 20–60 ng/mL of intact and bioactive plasmid DNA was released. Cells plated on the Cdk2i scaffold showed a decrease in Cdk2 mRNA expression by ~51% and 30% compared to the control and EGFPi scaffold, respectively. This translates to a 40% decrease in the proliferation activity of the MCF-7 breast cancer cell line, as well as the presence of an increased number of dead cells, based on the decreased Cdk2 mRNA expression, successfully demonstrating the delivery of bioactive RNAi-based plasmid DNA from an electrospun polymer scaffold. The main aim of disrupting cell-cycle regulation and suppressing the proliferation of cancer cells was achieved by inducing apoptosis.

Similarly, Chen et al. [114] studied the relationship between acidic/alkaline hydrolysis and a bulk/surface degradation mechanism to achieve the desired prolonged release required for efficient gene silencing. The results revealed the stability of the integrated chitosan/siRNA polyplex. Both pH 7.4 and pH 5.5 showed a triphasic release profile; however, pH 7.4 was based on bulk erosion, while pH 5.5 was a combination of bulk and surface erosion. Homogenous hydrolysis was observed following a short alkaline pre-treatment yielding a nearly zero-order release profile. siRNA transfection was then investigated, and the results revealed that the siRNA/chitosan encapsulated in the nanofibre showed 50% EGFP gene silencing 48 h post-transfection. In another scenario, Lee et al. [105] encapsulated an adeno-associated virus (a gene carrier) within electrospun nanofibres made up of a composition of blended mixtures of elastin-like polypeptides (ELP) and poly-(ε-caprolactone) (PCL), which was employed to transduce fibroblasts adherent on the nanofibres. When combined, the mechanical properties of ELP and PCL were significantly fabricated, providing the optimum tuneable properties to achieve a controlled release of the adeno-associated virus (AAV) vectors and robust cellular transduction on the nanofibres. The observed results prove that the combination of the ELP/PCL polymer cocktail and its ability to manipulate the release of AAV vectors from the nanofibres makes it a suitable delivery vehicle for tissue engineering.

In cancer studies, the complexity lies in the alteration of genetic and epigenetic mutations and the altered signalling pathways in tumour cells. The rationale behind developing electrospun nanofibres encapsulated with chemotherapeutics and gene–drug therapy in treating glioblastoma and various cancer is that it may be a potential clinical solution in treating glioblastoma. The use of electrospun nanofibres in cancer therapy thus far has shown promising results in circumventing drug or multi-drug resistance in cancer and undesired side effects and therapeutic outcomes through its ability to act as a localised treatment, therefore, increasing bioavailability and the desired pharmacodynamic response.

## 4. Delivery of Gene Therapy Drugs Using Electrospun Polymeric Nanofibres

In the treatment of GBM, we discuss the use of gene therapy drugs for the encapsulation of STING agonists embedded into polymeric nanofibres and the encapsulation of STING agonists in nanoparticles as one of the most radical technological approaches applied in the treatment of GBM. Gene delivery that does not require viral vectors has gained significant interest related to immunogenicity, the field of oncology and long-term side effects [136]. Gene therapy drugs are mainly divided into plasmid DNA, small interfering RNA, micro RNA and short hairpin RNA, as well as antisense oligonucleotides in the modulation of gene expression [22]. Gene therapy is still in its early stages, and it is used as an alternative for the prevention and cure of GBM or various different diseases that conventional therapy cannot mitigate. Therefore, it is significant that the barriers faced in gene delivery are identified and measures are put in place to circumvent drawbacks associated with gene therapy prior to the development of an appropriate gene–drug delivery vehicle (e.g., stability and serum–protein interactions, poor cell uptake and targeting, cell–cell recognition and reduced transfection) [137]. This can help narrow down the selection of an appropriate vehicle that is able to specifically target the cells of interest, preventing undesirable toxicities as well as ensuring the optimal therapeutic outcome.

Polymers in gene delivery have focused mainly on sustained delivery at the site of interest in tissue engineering, cancer therapy or the study of stem cells. Polymeric nanofibres fall under the non-viral vector category of a delivery system in gene therapy, as discussed above. The delivery of non-viral vectors such as naked plasmid DNA or DNA polyplexes as combinatory drug carriers has been encapsulated in electrospun nanofibres due to the ease of production of these nanofibres and the ability to ensure that the fibre remains intact in comparison to viral vectors [18]. However, for viral vectors, the viral genomic sequence is stripped and the gene of interest is packed into the viral capsid and further fabricated into electrospun nanofibres in an attempt to prolong gene expression and ensure efficient gene delivery [138]. The use of viral vectors, however, has been limited by safety issues, despite the effort to minimize side effects and immunogenicity issues. On the contrary, non-viral delivery systems have proven their ability to act as a targeted drug delivery vehicle, with low toxicity associated with the polymer of choice, minimal side effects and low cost of production [20]. The first non-viral gene delivery system was developed by Felgner et al. [139], where a cationic lipid-based system was fabricated for DNA transfection. It is hypothesized that the developed lipid-based delivery system formed an interaction with the negatively charged phosphate group that is present in nuclei acids via an electrostatic force, forming nanoparticles known as lipoplexes. The genetic information was preserved from the degradation of these lipoplexes and was able to target mammalian cells [140]. Other non-viral polymer delivery systems were discovered after this important breakthrough in the field of medicine.

Electrospun nanofibres in gene–drug delivery have gained popularity in terms of their versatility and functionalization, biodegradability, ease of synthesis and scalable production. In terms of the manufacture of nanofibres in gene delivery, electrospinning is versatile, as the composition of the polymer solution and its processing parameters establishes the mechanical, biological and kinetic properties of a nanofibre [133]. However, the two parameters that must be considered when electrospinning gene-encapsulated nanofibres are the effect of direct contact between the organic solvent and the bioactivities of gene vectors, and high voltage which must be limited to ensure that electrospun nanofibres are suitable vehicles for use in gene delivery [18]. There are two ways a gene vector can be encapsulated in electrospun nanofibres. One way is direct encapsulation of the gene vector during electrospinning. The direct encapsulation method, however, involves the mixing of the polymer and the gene via blending, emulsion and co-axial electrospinning, with the primary focus on achieving a sustained release of gene vectors. The second method involves the immobilization of the gene vectors on the surface of the fibres post-electrospinning. The reason for the immobilization of the gene vectors is to reduce the effects of organic solvents or a high voltage during the formation of the fibres on the gene vectors’ bioactivities—a relationship that extends to efficient gene delivery.

Klabukov et al. [141] subcutaneously implanted a fabricated modified polycaprolactone (PCL) microfibre scaffold by encapsulating pCMV-VEGF165-plasmid as a gene therapy drug in the intrascapular area of rats for angiogenesis evaluation and to study the relationship between vascularisation and degradation. The concentration variable of the plasmid was 0.005 ng per 1 mg of PCL, known as the LCGroup, and 0.05 ng per 1 mg, known as the HCGroup. The scaffolds were excised on days 7, 16, 33, 46 and 64 to evaluate the density and diameter of the vessels and the diameter of the microfibre. Based on their observations, the increase in the number of vessels was observed among all groups, as was scaffold resorption. Day 33 showed a 42% increase in the vascular density of the HC group in comparison to the control group. Enhanced angiogenesis in the HC group confirmed that the effect is dependent on the pCMV-VEGF165-plasmid dose. No significant statistical difference was observed between vascular growth and scaffold degradation. These results confirm that the fabrication of PCL by encapsulation of the pCMV-VEGF165 plasmid improved vascularisation 33 days post-implantation; however, vessel growth and degradation rate were not correlated.

### Encapsulation of STING Agonists for Tumour Regression in GBM

STING is a protein-coding gene situated in the endoplasmic reticulum and is used as a critical sensor or target for therapeutics for infectious diseases and cancers [142,143,144]. The activation of the stimulator of interferon genes (STING) pathway in the modulation of the immune system has gained much attention for immunosuppressive tumours such as GBM; however, they have been associated with high toxicity and premature degradation, causing limited efficacy [145]. The immune checkpoint blockade (ICB) has shown remarkable anti-cancer properties and, in some cancers, can be re-tuned to fight against tumour formation [146]. Studies have shown that these properties are made possible by moulding a TME to be more conducive to the activation of the immune system by the targetting of tumours that are resistant to ICB [142,147]. The term ‘cold’ is usually used to describe GBM tumours due to the difficulty of the immune system in being able to detect and target these tumours, allowing them to proliferate unchecked, regardless of the diverse immunotherapy drugs available. In GBM, the ‘cold’ TME of GBM has proven to be influenced by the DNA methylation pattern in the promoter region of STING, where it contributes to immunosupression [146]. Therefore, the rationale behind the activation of the STING pathway is to remodel the brain TME, producing an anti-tumour effect by activation of the innate immune cells and the body’s natural killer cells.

This response is dependent on the function of the myeloid cells, which is cardinal in GBM detection. However, in GBM, myeloid cells are re-modelled within the TME, where the abundance in the TAMC composition provides an optimal contribution to the immuno-suppressiveness of GBM, therefore contributing to the lack of treatment efficacy in GBM, which includes radiotherapy, chemotherapy and immunotherapy [102,148,149]. This relationship extends to the resistance of GBM tumours to ICB due to the highly immunosuppressed ‘cold’ tumours in GBM which lack infiltrative T-cells but are rich in immunosuppressive tumour-associated myeloid cells (TAMCs). The purpose of STING in this case is to increase the infiltrative T-cells via the pro-inflammatory activation of the tumour stroma in the immunologically ‘cold’ tumours to alter the myeloid-deprived suppressor cells of its suppressive phenotype [142]. For example, the activation of STING can remodel the M2 tumour-supportive macrophages towards the pro-inflammatory M1 phenotype and is also used to prime the cytotoxic T-cells against the tumour antigens [150]. Motivated by the advantages of the activation of STING, the use of polymeric systems to encapsulate STING agonists to activate the STING pathway for the treatment of GBM has been explored by various researchers.

To leverage this, Zhang et al. [149] fabricated a bridging lipid nanoparticle (B-LNP) to prime TAMCs to GBM cells via anti-CD47/PD-L1 dual litigation. The observed results showed that the engager B-LNP suppresses the CD47/PD-L1 while simultaneously encouraging the phagocytic activity of TAMC. Post-tumour engulfment, the B-LNPs were further fabricated to encapsulate a non-nucleotidyl agonist as a stimulator of interferon genes (diABZi). A transcriptomic and metabolic switch in TAMCs was induced by the fabricated B-LNPs encapsulated with diABZi used for in vivo treatment, causing the immunosuppressive cells to switch to antitumour effectors, therefore causing T-cell infiltration and activation in brain tumours. The fabricated B-LNP/diABZI nanoparticles were then administered in pre-clinical murine models, and treatment was used in combination with radiotherapy to induce brain tumour regression and to influence immune memory against glioma. In another scenario, Wilson et al. [151] encapsulated cyclic dinucleotides (CDNs) via a biodegradable poly(beta-amino ester) (PBAE) for in vitro delivery of the CDN to the cytosol, causing an immune response at >100-fold lower than extracellular CDN concentrations. The CDN-PBAE nanoparticle produced a log-fold potency improvement in treating B16-type melanoma tumours when combined with the PD-1-blocking antibody in comparison to unencapsulated CDN.

In a similar manner, Yang et al. [152] fabricated a nanoparticle-encapsulated bacterial-derived cyclic dimeric adenosine monophosphate (CDA) in nanoscale coordination polymers (self-assembling polymer), forming a STING agonist known as ZnCDA. Based on the route of administration, ZnCDA was intravenously injected, prolonging CDA circulation as well as specifically targeting brain tumours and exerting an anti-tumour effect based on a single dose in an abundance of pre-clinical cancer models. Based on their findings, ZnCDA disrupts endothelial cells in the tumour vasculature, causing tumour accumulation. The activation of ZnCDA targets tumour-supportive macrophages to regulate antigen processing and presentation to prime the cytotoxic T-cells against these antigens. This causes a knock-on effect of radiotherapy and ICBs by re-instating the anti-tumour activity in immunologically ‘cold’ glioma tumours, exerting a promising therapeutic response in resistant GBM tumours. Wang et al. [145] fabricated a self-assembling supramolecular hydrogel by encapsulating the cyclic-d-AMP STING agonist which was electrostatically complexed with nanotubes, comprising a peptide–drug conjugate, a peptide that binds to the protein neuropilin-1, which is highly expressed in tumours, and the chemotherapeutic agent camptothecin. The encapsulated supramolecular hydrogel was then locally applied in multiple murine tumours in mouse models, and the observed results showed that a single dose of the STING-agonist-induced tumour regression increased animal survival and produced a long-term immunological memory response. These helped to prevent tumour recurrence in mice and metastasis adjacent to the primary site.

Motivated by this, Li et al. [144] prolonged the activation of the innate immune pathway by encapsulating a polyvalent STING agonist in a pH-sensitive polymer with a seven-membered ring with a tertiary amine (PC7A) where the polymer formed a STING-PC7A condensate. The polymer–agonist combination was applied to subcutaneous tumour-bearing mice and resected human tumours and lymph nodes, and showed that PC7A induces a more prolonged formation of pro-inflammatory cytokines compared to the natural STING ligand by binding to a non-competitive STING surface. In response to this, an anti-tumour effect is produced which is dependent on STING expression of CD8^+^ T-cell activity leading to a synergistic therapeutic outcome, suggesting new therapeutic opportunities in GBM treatment. Herein, we have explained how the activation of the STING pathway has been proven to remodel the TME and attract innate immune cells for anti-tumour effects in mice models, producing a long-term memory in the immune system. To date, GBM remains resistant to therapy that has shown promising effects in other cancers. Therefore, the goal of GBM therapy is to remove the shield that the tumour is using to protect itself by the use of implanted drug-loaded polymers by remodelling the TME to deliver a combination of therapies.

## 5. Conclusions, Challenges and Future Perspectives

### 5.1. Challenges

Drug- and gene-loaded electrospun nanofibres are rapidly revolutionizing the field of drug delivery associated with GBM in both academic and clinical practices, and may hold the potential to tackle the inherent challenges associated with GBM therapy. ES-loaded nanofibres require less drug incorporation in electrospun nanofibres due to the tuneable properties of GBM therapy, decreasing unwanted side effects, lowering toxicity, increasing the surface area to volume ratio, and giving long-lasting and curative benefits, to name a few. A substantial amount of effort and financial support has been dedicated to the advancement of research using ES nanofibres in the treatment of GBM therapy from basic to clinical-scale research, as reported in this review, showing how rapidly the interest in this treatment is evolving and its potentially extensive application in both academic and clinical practices [22]. As of 2022, the FDA has approved two new drugs for GBM–Dabrafenib and Trametinib—in addition to the four pioneering gold-standard drugs outside of TMZ and one device—lomustine, intravenous carmustine, carmustine wafer implants, bevacizumab (BVZ), and tumour treatment fields (TTFields) [115]. Gene therapy has 23 gene therapy drug products for treating various diseases approved by the FDA [22,153].

However, these treatments are hampered by unwanted side effects, toxicity issues, and delivery efficacy, requiring a potent gene and drug delivery system that can circumvent issues related to conventional therapy. The ability to use different electrospinning techniques, including co-axial, emulsion, and blend electrospinning, to encapsulate hydrophobic, hydrophilic and non-viral gene vectors may be a potential clinical solution in the treatment of GBM and various cancers. Incorporating these different types of drugs and gene therapy ensures the drug or gene is protected from the surrounding systemic environment, ensuring that a maximum therapeutic response is achieved at the site of action in a controlled manner. However, the challenge remains in commercializing these electrospun nanofibre therapies in GBM. A significant gap exists between clinical investigation and commercialization in the pharmaceutical market, accounting for no evidence of drug-loaded or gene-loaded electrospun nanofibres approved by the FDA [22]. This remains a challenge to date in the clinical development of these carriers. The ongoing limitations in human and animal models concerning the TME may vary in therapeutic efficacy due to the inaccuracy in mimicking the tumour microenvironment or solid tumours. The inability to recapitulate the tumour microenvironment due tumour heterogeneity (genotype and phenotype), which is related to the electrospun nanofibre’s morphology and physico-chemical properties, is a challenge that has to be addressed.

From an economic point of view, the low yield of electrospinning equipment also affects the reproducibility and the ability to scale up manufacturing processes, and tremendous is effort required to resolve this issue, for example, issues with regard to electrospinning processing parameters, such as electrostatic charges, loss of sample, design optimization and the compatibility of these drug- and gene-loaded nanofibres post-implantation. These shortcomings limit these nanofibres to preclinical studies, making it imperative for a viable strategy to be developed to progress these fabricated nanofibres from bench/academic research to clinical trials [56]. It is also imperative to publish negative data when studies enter in vivo trials, as this may help other scientists tailor ongoing research to increase the chance of success of these drug-loaded and gene-loaded nanofibres in treating GBM or any other types of disease.

### 5.2. Future Perspectives

Despite the ongoing challenges, electrospun drug/gene-loaded nanofibres have proven to be a potent drug delivery system and have significantly progressed in treating GBM. Notably, in the study of GBM, the complexity lies in the alteration of genetic and epigenetic mutations and the signalling pathways that are altered in tumour cells. Therefore, the use of drug and gene therapy drugs embedded in polymeric electrospun nanofibres is one of the most radical technological approaches to addressing these mutations in glioblastoma. Designing electrospun nanofibres with tailored mechanical properties to encapsulate drug or gene therapy in treating GBM and different cancers may be a potential clinical solution. Electrospun nanofibres have shown promising results in circumventing drug or multidrug resistance in cancer and undesired side effects, and act as a multi-functional strategy with the ability to act as a localised treatment. These results increase bioavailability and the desired pharmacodynamic response. Our team is also actively involved in the research of different drug-loaded nanofibres and nanoparticles in treating different grades of brain tumours and different types of cancers [116,117,118].

The research on electrospun nanofibres is moving forward at an incredibly fast pace owing to their applications in diverse domains. To the best of our ability, we have highlighted the advantages of electrospun nanofibres in treating GBM from physico-chemical properties, mimicking the extracellular matrix of the tumour microenvironment to understand the influence of chemistry and mechanics on GBM behaviour to sustain the release of a drug or gene–drug therapy on the road to recovery. These influences are significant in exerting maximal therapeutic effects when designing these electrospun fibres as novel devices. In the future, these electrospun nanofibres will require a path to clinical trials to understand the safety and efficacy of these treatments for long-term use in patients with GBM. 

## Figures and Tables

**Table 1:** Mechanism of drug release based on the type of drug delivery system.

Polymer Drug Delivery System	Drug	Mechanism of Drug Release	Cancer Cell Type	Ref.
Poly(ε-caprolactone) (PCL)/ gelatin (GT)	SN-38 97-ethyl-10-hydroxy camptothecin)	Diffusion and anomalous transport	Human glioblastoma 251 and U87 cells	[81]
Poly(ethylene glycol)– poly(l-lactic acid) (PEG–PLLA)	1,3-bis(2- chloroethyl)-1-nitrosourea (BCNU)	Diffusion/Degradation of polymer matrix	Glioma C6	[82]
Poly-(d,l-lactide-co-glycolide) (PLGA)	Paclitaxel (PTX)	Polymer matrix degradation	Glioma C6 cells in rats	[83]
Poly(l-actide) (PLA)/poly- (d,l-lactide-co-glucolic acid) (PLGA)	Cisplatin (CP)	Diffusion	Rat C6 glioma cells	[84]
Poly(ethylene glycol)-(llactic acid) (PEG–PLA)	Paclitaxcel (PTX) and Doxorubicin Hydrochloride	Diffusion	Murine glioma c6 cells	[85]
Polypropylene carbonate Ca Alginate MPs	Paclitaxcel (PTX) and Temozolomide (TMZ)	Prolonged release/Polymer matrix degradation	Glioma C6 cells in rats	[86]
poly (ε-caprolactonediol) (PCL)/Polyurethane(PU)	Temozolomide (TMZ)	^1^ Diffusion	U87 Cells	[87]
PLGA-PLA-PCL blends	Temozolomide (TMZ)	Sustained Release/Polymer matrix degradation	U87 cells and rat c6 glioma cells	[88]
poly(lactic acid) (PLA)/polyethylene oxide (PEO)	Rapamycin	Sustained Release/Polymer matrix degradation	Human glioblastoma 251 and U87 cells	[89]
Poly-(d,l-lactide-co-glycolide) (PLGA)	1,3-bis(2- chloroethyl)-1-nitrosou)rea (BCNU)	Polymer matrix degradation	Wistar Rats	[90]
poly(ε-caprolactone) (PCL)/polyvinylpyrrolidone (PVP)	Mycophenolic Acid	N/A	U87 cells	[91]
poly (ε-caprolactone)	Daunorubicin	Polymer matrix degradation	U87 cells and Hela	[92]
poly(L-lactic acid) (PLLA)	Doxorubicin	Initial Rapid Release followed by sustained release	Hela Cells	[9]
poly(ε-caprolactone)-poly(ethylene glycol)-poly(ε-caprolactone)	Curcumin	Diffusion	Glioma 9 L	[93]
Poly-(d,l-lactide-co-glycolide) (PLGA)/polyethylenimine (PEI)	Paclitaxcel (PTX)	Sustained Release	BALB/c nude mice	[94]
polycaprolactone (PCL)/gelatin (Gel)	bacterial cellulose nano-crystal (BCNC)	N/A	U251 MG	[95]

^1^ Note: N/A = Not available; PTX = Paclitaxcel; TMZ = Temozolomide; DXR Doxorubicin; DOX = Doxorubicin hydrochloride; CP = Cisplatin; BCNU = 1,3-bis(2-chloroethyl)-1-nitrosourea; PCL = Poly(ε-caprolactone); PEG-PLLA = Polyethylene glycol-poly(L-lactic acid); PLA = Polylactic acid; PEO = Polyethylene oxide; GEL = Gelatin; PLGA = Poly-(D, L-lactide-co-glycolide); PVP = Polyvinylpyrrolidone; PEG polyethylene glycol.
